# Synergistic action of phages and lytic proteins with antibiotics: a combination strategy to target bacteria and biofilms

**DOI:** 10.1186/s12866-023-02881-2

**Published:** 2023-05-23

**Authors:** Han Lu, Zong Li, Amro Elbaz, Shou-Qing Ni

**Affiliations:** 1 Shandong Provincial Key Laboratory of Water Pollution Control and Resource Reuse, Shandong Key Laboratory of Environmental Processes and Health, School of Environmental Science and Engineering, Shandong University, Qingdao, 266237 Shandong China; 2 College of Recourses and Environment, University of Chinese Academy of Sciences, Beijing, 101408 China; 3 Environmental Engineering Department, Zagazig University, Zagazig City, 44519 Egypt

**Keywords:** Phages and lytic proteins, Antibiotic, Synergistic sterilization

## Abstract

**Background:**

Multidrug-resistant bacteria continue to emerge owing to the abuse of antibiotics and have a considerable negative impact on people and the environment. Bacteria can easily form biofilms to improve their survival, which reduces the efficacy of antibacterial drugs. Proteins such as endolysins and holins have been shown to have good antibacterial activity and effectively removal bacterial biofilms and reduce the production of drug-resistant bacteria. Recently, phages and their encoded lytic proteins have attracted attention as potential alternative antimicrobial agents. The aim of the present study was to investigate the sterilising efficacy of phages (SSE1, SGF2, and SGF3) and their encoded lytic proteins (lysozyme and holin), and to further explore their potential in combination with antibiotics. To the ultimate aim is to reduce or replace the use of antibiotics and provide more materials and options for sterilisation.

**Results:**

Phages and their encoded lytic proteins were confirmed to have great advantages in sterilisation, and all exhibited significant potential for reducing bacterial resistance. Previous studies on the host spectrum demonstrated the bactericidal efficacy of three Shigella phages (SSE1, SGF2, and SGF3) and two lytic proteins (LysSSE1 and HolSSE1). In this study, we investigated the bactericidal effects on planktonic bacteria and bacterial biofilms. A combined sterilisation application of antibiotics, phages, and lytic proteins was performed. The results showed that phages and lytic proteins had better sterilisation effects than antibiotics with 1/2 minimum inhibitory concentrations (MIC) and their effect was further improved when used together with antibiotics. The best synergy was shown when combined with β- lactam antibiotics, which might be related to their mechanism of sterilising action. This approach ensures a bactericidal effect at low antibiotic concentrations.

**Conclusions:**

This study strengthens the idea that phages and lytic proteins can significantly sterilise bacteria in vitro and achieve synergistic sterilisation effects with specific antibiotics. Therefore, a suitable combination strategy may decrease the risk of drug resistance.

## Background

Pathogens are widely distributed in the environment and pose a threat to public health. Antibiotic abuse is responsible for the emergence and persistence of antimicrobial-resistant bacteria [1]. The widespread presence of antimicrobial-resistant bacteria increases the difficulty of controlling bacterial pollution and has a huge negative impact on human and ecological environments. In addition to abundant planktonic flora, bacteria tend to form biofilms on biotic and abiotic surfaces to facilitate their survival and better adapt to the environment [2]. Biofilms are mainly composed of mucus secreted by the bacteria that envelopes them. With high resistance and low permeability to typical antimicrobial drugs, biofilms not only protect the bacteria from the host immune cells but also reduce the efficiency of antibiotic-based treatment, which in turn increases the risk of antibiotic resistance development [3, 4]. The problem of the "failure" of antibiotics against biofilms and continuous enhancement of bacterial resistance are becoming increasingly prominent. To effectively address these issues, phages and their encoded lytic proteins have recently been investigated as potential alternative antimicrobial agents.

Notably, phages and their encoded lytic proteins can efficiently infect planktonic bacteria, and various studies have reported the effectiveness of phage cocktails and lytic proteins for biofilm destruction [5–8]. Compared with individual phages, phage cocktails have more widespread applications owing to their broad effect against bacteria, including the clinical treatment of multidrug-resistant bacterial infections and the control of multiple bacterial infections in poultry products, food, and sewage systems [9–12]. Lytic proteins encoded by phages, such as endolysins [13], holins [14], and depolymerases [15], act on the bacterial peptidoglycan skeleton and cell membrane. Studies have confirmed that these lytic proteins can rapidly penetrate Gram-positive bacteria and eventually cause cell death when added extracellularly. They can also disrupt biofilm structures by killing bacteria in the biofilm matrix [16]. However, in Gram-negative bacteria, peptidoglycans are protected by the outer membrane layer; therefore, the potent bactericidal activity of these proteins is generally limited. Swapnil et al. [15] demonstrated that phage-encoded depolymerases act against biofilms formed by *E. coli*. The few proteins that can penetrate Gram-negative bacteria are also less susceptible to drug resistance than antimicrobial agents, such as recombinant lysin, which can kill persisters of *Pseudomonas aeruginosa* that survive ofloxacin treatment [17]. This establishes a foundation for the use of phages and their encoded lytic proteins to treat bacteria and biofilms.

Phages and their encoded proteins are of great value as alternatives to antibiotic treatments. Moreover, combining them with other antimicrobial agents can not only overcome the drawbacks of unstable protein applications, but also remove bacteria and biofilms more efficiently [18–21]. Similar to phage cocktails, phage lytic proteins combine with other antimicrobials, such as antibiotics, to target different receptors. Three phages, SSE1, SGF2, and SGF3, obtained in a previous study [22–24] all had cleavage activity against *Shigella spp*. The encoded lytic proteins LysSSE1 and HolSSE1 were annotated as lysozyme and holin, respectively, based on the NCBI database alignment. They have been shown to have an extensive cleavage spectrum with lytic activity against various bacteria, including *Shigella*, *Escherichia coli* and *Staphylococcus aureus*.

In this study, different phages (SSE1, SGF2, and SGF3) along with antibiotics and lytic proteins (LysSSE1 and HolSSE1) were used to investigate their ability to resist bacteria and biofilms. Different classes of antibiotics were selected to analyse their synergy with phages and proteins. We used combination of these bactericidal substances to determine the best sterilisation method, reduce the use of antibiotics, and maximise the sterilisation effect. We further analysed the potential interactions between sterilisation methods. This provides a new strategy for targeting planktonic bacteria and bacterial biofilms. Our results highlight novel materials and methods for future bacterial infection control and the value of phages and lytic proteins.

## Methods

### Bacterial strains, phages, and lytic proteins

Phages SSE1, SGF2, and SGF3 were isolated from *S. dysenteriae* 1.1869, *S. flexneri* 1.1868, and *S. flexneri* 1.10599, respectively [22–24]. They were all stored in -80℃ mixed with glycerol. Details of the phages (Table 1a) and bacterial strains (Table 1b) used in this study are summarised in Table 1. LysSSE1 and HolSSE1 were used as the lytic proteins; they are a lysozyme and holin protein, respectively, encoded by the phage SSE1 and have been extracted and identified in a previous study [25]. All bacterial strains were purchased from the two major preservation platforms, Chinese Center for Disease Control and Prevention (Chinese CDC) and China General Microbiological Culture Collection Center (CGMCC). After activated in nutrient broth (NB) medium (Hopebio, 1% peptone, 0.3% beef powder, and 0.5% NaCl) at 37℃, these strains were stored in 20% glycerol at -80℃ until further use.

**Table 1:** Phage details and bacterial strains sources used in the study

**a Details of phages**			
**Characteristics**	**SSE1**	**SGF2**	**SGF3**
Host bacteria	*Shigella dysenteriae* 1.1869	*Shigella flexneri* 1.1868	*Shigella flexneri* 1.10599
Morphological features	*Myoviridae*	*Phoviridae*	*Microviridae*
Nucleic acid	169,744 bp	76,964 bp	5,386 bp
Family	*Tevenvirinae*	*Kuravirus*	*Sinsheimervirus*
Infectable bacteria	*Shigella dysenteriae 1.1869* *Shigella flexneri 1.10599* *Shigella baumannii 1.10618*	*Shigella flexneri 1.1868*	*Shigella flexneri* 1.10599
**b Type of the bacterial strains**			
**Strain Type**		**Source**	
* Shigella dysenteriae 1.1869*		CGMCC	
* Shigella flexneri 1.10599*		CGMCC	
* Shigella flexneri 1.1868*		CGMCC	
* Shigella baumannii 1.10618*		CGMCC	
* Escherichia coli EDL933*		Chinese CDC	
* Staphylococcus aureus 1.8721*		CGMCC	
* Staphylococcus aureus 1.2875*		CGMCC	

*CDC* Chinese Center for Disease Control and Prevention, *CGMCC* China General Microbiological Culture Collection Center

### Selection of antibiotics and determination of minimum inhibitory concentrations (MICs)

Different types of antibiotics have different bactericidal mechanisms. Nine antibiotics were selected for this study based on their classification and mechanism of action. The categories and sites of action are listed in Table 2. Antibiotic dissolution was configured to an appropriate concentration using the corresponding solvent. The MICs were determined according to the standards of the National Standardization Committee of Clinical Laboratory Standardization (NCCLS) [26]. NB medium was first added to a 96-well plate and the drug was then added to the wells for magnified dilution to achieve final antibiotic concentrations of 128, 64, 32, 16, 8, 4, 2, 1, 0.5, 0.25, 0.125, and 0.06 µg/mL. Host bacteria with a titre of 10^6^–10^7^ CFU/mL were then added and cultured at 37℃ overnight. The absorbance was measured at 600 nm using an enzyme-labelled instrument (SynergyTMH1, BioTek). The MIC was the minimum concentration that inhibited the growth of the host bacteria. These results were interpreted in combination with the NCCLS criteria.

**Table 2:** MIC of antibiotics against *Shigella*

Antibiotic	Type	Action site	MIC^a^ (µg/mL)		
			*S. dysenteriae* CGMCC 1.1869	*S. flexneri* CGMCC 1.1868	*S. flexneri* CGMCC 1.10599
Erythromycin	macrolides	tRNA	0.125 (S)	4 (I)	0.06 (S)
Gentamicin Sulphate	aminoglycoside	ribosomal 30S subunits	0.25 (S)	8 (R)	0.125 (S)
Chloramphenicol	amyl alcohol	peptide linkage	0.06 (S)	0.06 (S)	0.06 (S)
Cefotaxime	β- lactam	cell wall	0.06 (S)	0.06 (S)	0.06 (S)
Cefoxitin	β- lactam	cell wall	0.06 (S)	2 (S)	0.06 (S)
Cephalothin	β- lactam	cell wall	0.06 (S)	0.125 (S)	0.06 (S)
Cardelmycin	aminocoumarin	DNA gyrase	0.125 (S)	8 (R)	0.125 (S)
Tetracycline hydrochloride	tetracyclines	rRNA	0.06 (S)	0.06 (S)	0.06 (S)
Polymyxin B sulfate	polypeptides	LPS	0.125 (S)	2 (S)	0.06 (S)

^a^S, I, and R represented the degree of strain response to each antibiotic, with sensitivity, intermediary and resistance, respectively

### Removal of planktonic bacteria by phage and lytic protein combined with an antibiotic

To evaluate the combined effect of the phages and antibiotics on the inhibition of planktonic host bacteria, 100µL bacteria at 10^7^ CFU/mL were added to 96-well plates, followed by 100µL phages at 10^7^ to 10^8^ PFU/mL. The corresponding antibiotics were added to the experimental groups at a final concentration of 1/2 MIC. The control groups were treated separately with either no phages or antibiotics, only phages, or only antibiotics. Three parallel samples were used for each group. After the mixture was incubated overnight at 37 ℃, the absorbance at 600 nm (OD 600) was measured using an enzyme-labelled instrument. The A600 values were used to determine the relative removal rates of host bacteria. The relative A600 was calculated as follows: A600 = OD 600 (added phage/antibiotic) / OD 600 (host bacteria).

Bacteria at a titre of 10^7^ CFU/mL were transferred to a 96-well plate to test the inhibitory effects of lytic proteins and antibiotics on planktonic bacteria. LysSSE1 and HolSSE1 at individual final concentrations of 60–65 mg/mL, antibiotic (final concentration of 1/2 MIC), and lytic protein combined with antibiotic (1/2 MIC) were added. The control group was not treated with the lytic proteins or antibiotics. OD 600 was measured after 2 h of incubation at 37℃. Three parallel measurements were performed for each group. The relative removal rate of bacteria was calculated as A600 = OD 600 (added lytic protein/antibiotic) / OD 600 (bacteria).

### Removal of the bacterial biofilms by phage and phage cocktails

Given the difficulty of bacterial biofilm removal, 200µL phage cocktail was used. Host bacteria at 10^7^ CFU/mL titre, were added to 96-well polystyrene plates and incubated for 24 h at 37℃. After biofilm formation, all wells were washed with PBS. After bacterial biofilm fixation, the experimental groups were treated with a phage or phage cocktail (200µL, titre of 10^7^ to 10^8^ PFU/mL), and the control groups were treated with equivalent volume of sterile PBS. Each well was then washed with PBS and stained with 1% crystal violet (200µL) for 30 min after incubation for 24 h at 37℃. The dye was removed, and the wells were cleaned with PBS. Subsequently, biofilms were dissolved with 200µL of 95% absolute ethanol after drying at room temperature. OD 570 was measured using an enzyme-labelled instrument, with three biological replicates in each group. The relative removal rate of the bacterial biofilms was calculated as follows: A 570 = OD 570 test / OD 570 control (PBS only).

### Removal of single and multiple biofilms by lytic proteins with an antibiotic

Multiple bacterial biofilms were introduced to study the removal ability and application potential of the lytic proteins. Bacterial biofilms were cultured and fixed, as described in the previous section. For this analysis, the culture of multiple bacterial biofilms was performed using mixed suspensions of multiple bacteria, with each bacterial concentration of 10^7^ CFU/mL, 67µL. The tested groups were treated with LysSSE1 / HolSSE1 (60 mg/mL – 65 mg/mL), antibiotics (1/2 MIC), or a combination of lytic proteins and antibiotics. The control group was treated with PBS only. OD 570 was measured using an enzyme-labelled instrument after crystal violet staining and absolute ethanol solubilisation. Experiments were performed using three biological replicates. The relative removal rate of bacterial biofilm was expressed by A 570 as follows: 570 = OD 570 (lytic protein/antibiotic) / OD 570 (PBS only).

## Results

### MIC of the antibiotics

The MIC of various antibiotics against the three *Shigella* strains are shown in Table 2. Nine antibiotics were selected, based on the type of antibiotic and sterilisation method, and their antibacterial capacities against *Shigella* were determined. A final concentration of 1/2 MIC was used in combination with the phage and lytic proteins to remove bacteria in later experiments.

### Inhibition effects of phages and antibiotics on planktonic host bacteria

**Fig. 1:**
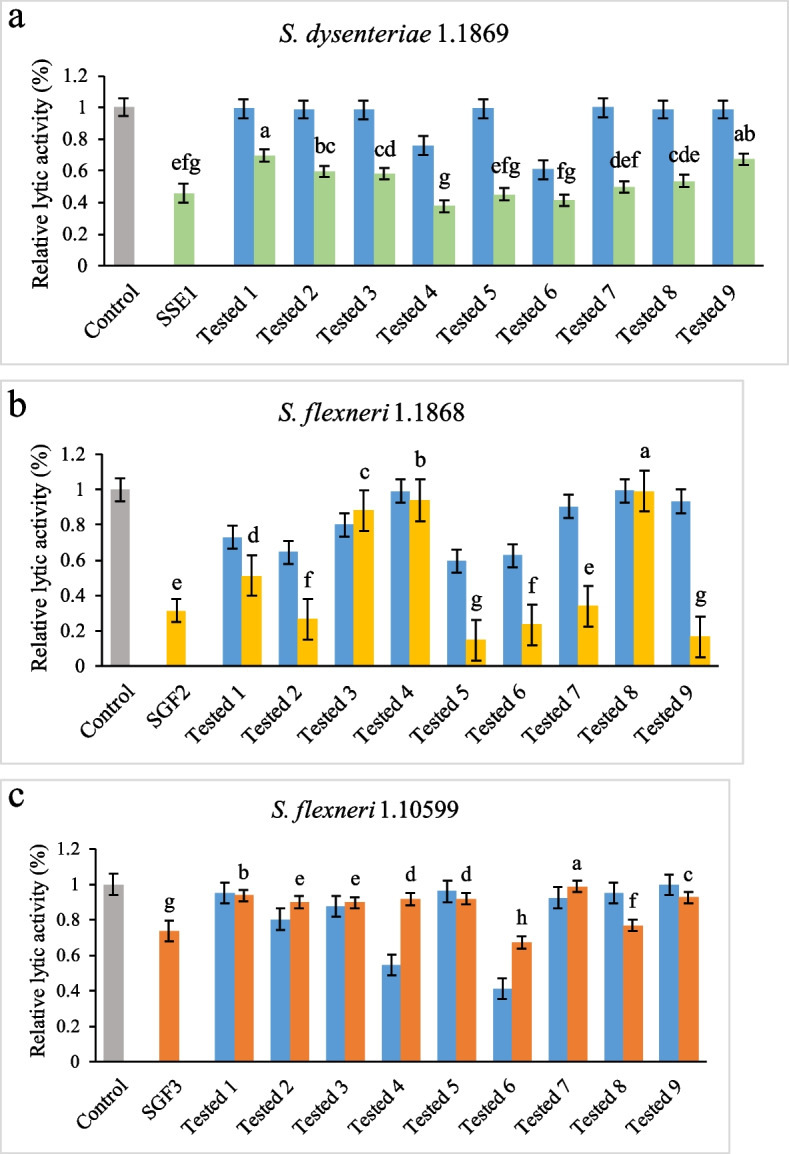
Phages were combined with antibiotics to remove different planktonic *Shigella*. **a** *Shigella* 1.1869 were removed by phage SSE1 and antibiotics, **b** *Shigella* 1.1868 were removed by phage SGF2 and antibiotics, **c** *Shigella* 1.10599 were removed by phage SGF3 and antibiotics. Gray represents negative control without phage and antibiotics, green represents phage SSE1 and its antibiotic combinations, yellow represents phage SGF2 and its antibiotic combinations, orange represents phage SGF3 and its antibiotic combinations, and blue represents antibiotic alone. Groups 1, 2, 3, 4, 5, 6, 7, 8, and 9 correspond to erythromycin, gentamicin sulphate, chloramphenicol, cefotaxime, cefoxitin, cephalothin, cardelmycin, tetracycline hydrochloride, and polymyxin B sulphate, respectively. The concentration of all the antibiotics used was 1/2 MIC. Error bars represent standard deviation of three biological replicates. The letters a, b, c, d, e, f, g, and h indicate significant results; the significance level was set at *p* < 0.05

Phages SSE1, SGF2, and SGF3 were used in combination with nine antibiotics to remove host *Shigella* strains, and the results are shown in Fig. 1. The titre of the phage used in each group was consistent, and the concentration of the antibiotics was 1/2 MIC. Figure 1a shows the sterilisation effects of phage SSE1 in combination with antibiotics. After overnight culture of phage SSE1 and antibiotics together with *S. dysenteriae* 1.1869, both phages and antibiotics effectively inhibited the growth of host bacteria. The removal rate of host bacteria by SSE1 alone was approximately 54.2%, and antibiotics also had a certain degree of inhibition of the strains; among these cephalothin had the best removal rate of approximately 39.3%. This demonstrated that the antibacterial removal of antibiotics alone was considerably less efficient than that of the phages. Comparison of the combined sterilisation effects showed that when antibiotics and phages were added together, the sterilisation effect was considerably higher than that of the antibiotics alone, and the removal rate was almost twice that of the antibiotics alone. When SSE1 was combined with cefotaxime, cefoxitin, and cephalothin, the removal rates of *S. dysenteriae* 1.1869 were 62.3%, 54.8%, and 58.7%, respectively, which were higher than those of phage SSE1 alone. The effects of the three sterilization methods on the removal of *S. dysenteriae* 1.1869 were significant different, and the combination of SSE1 and cefotaxime was the most favourable. Therefore, to better control *S. dysenteriae* 1.1869 contamination, both SSE1 and cefotaxime at 0.03 µg/mL are recommended.

The removal of planktonic *S. flexneri* 1.1868 is shown in Fig. 1b. Phage SGF2 exhibited a best sterilisation effect, with a removal rate of 68.5%. Cefoxitin was the most effective sterilising agent with a removal rate of approximately 40.4%. When phage SGF2 was combined with antibiotics, the sterilisation rate of the other seven groups, except for chloramphenicol and tetracycline hydrochloride, was more than half that of antibiotics alone, which showed better synergy. Moreover, the sterilisation effect of SGF2 with gentamicin sulphate, cefoxitin, cephalothin, and polymyxin B sulphate was better than that of SGF2 alone. Comparative analysis showed that the optimal combination was SGF2 and cefoxitin, with a removal efficiency of 85.1%. For the removal of *S. flexneri* 1.1868, it is recommended to use 1 µg/mL cefoxitin and SGF2 in combination for the maximal antibacterial effect.

Sterilisation with phage SGF3 was ineffective (Fig. 1c). The removal rate of *S. flexneri* 1.10599 with phage SGF3 alone was approximately 26.4%, compared to 45.3% and 58.9% with cefotaxime and cephalothin, respectively. This revealed that the antibiotics performed better than SGF3 did. Unlike SSE1 and SGF2, the antibacterial effect was not enhanced when phage SGF3 was used in combination with antibiotics. Both cefotaxime and cephalothin alone showed better removal effects on *S. flexneri* 1.10599. The best effect was achieved with 0.03 µg/mL cephalothin, which could be used to control *S. flexneri* 1.10599.

### Effects of phage and cocktails on host bacterial biofilm removal

**Fig. 2:**
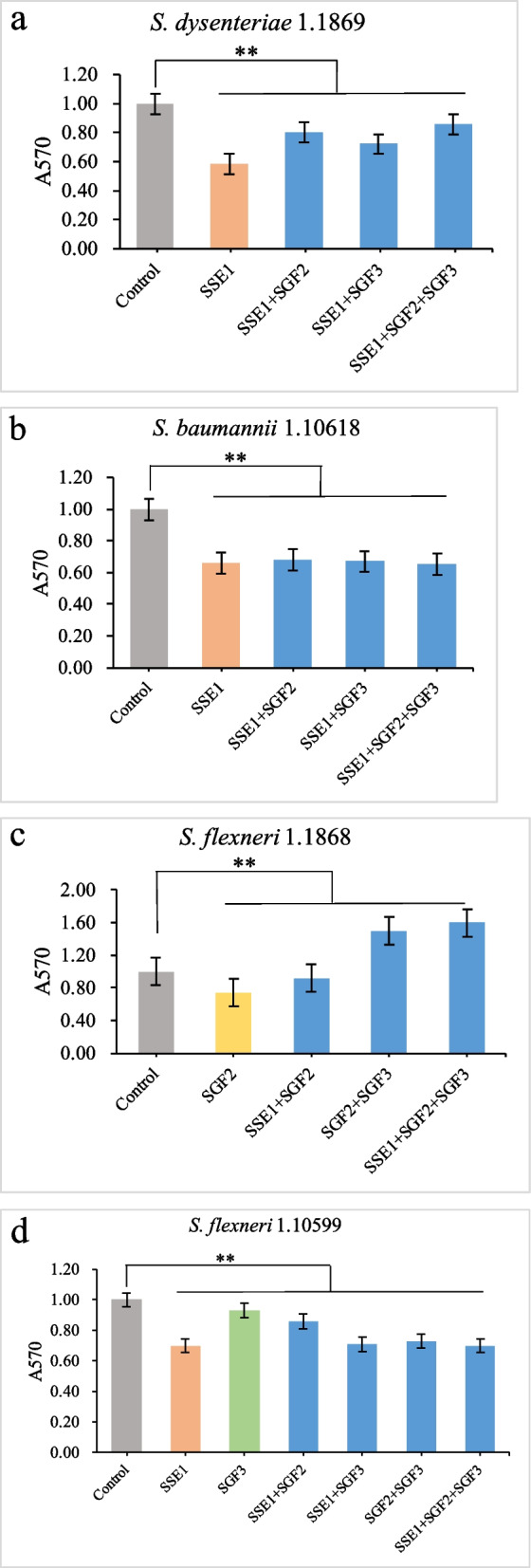
Removal effect of different bacterial biofilms by phages and cocktails. **a** *S. dysentery* 1.1869 biofilm was treated with phage SSE1 and its cocktails. **b** *S. baumannii* 1.10618 biofilm was treated with phage SSE1 and its cocktails. **c** *S. flexneri* 1.1868 biofilm was treated with phage SGF2 and its cocktails. **d** *S. flexneri* 1.10599 biofilm was treated with phage SSE1, SGF3, and their cocktails. Gray indicates control groups without phage addition, orange indicates only phage SSE1, yellow indicates only phage SGF2, green indicates phage SGF3 alone, and blue indicates phage cocktails of several different combinations. Error bars represent standard deviation of three biological replicates. Statistical analysis was performed using a paired sample t-test, and double asterisks indicate *p* < 0.01

Phage cocktails have been used to remove bacterial biofilms that are difficult to remove and expand the range of bacteria that can be targeted. To ensure accurate quantification, the total volume of the phage cocktail was the same as that used for a single phage. As shown in Fig. 2a, phage SSE1 alone performed better than the phage cocktail combinations for biofilm removal of the host bacterium *S. dysenteriae* 1.1869. The removal efficiency of phage SSE1 was 42.05%, and those of phages SSE1 + SGF2, SSE1 + SGF3, and SSE1 + SGF2 + SGF3 were 19.95%, 28.21%, and 13.85%, respectively, which were significantly lower than those of SSE1 alone. This indicated that these three phages were not synergistic in removing the biofilm of *S. dysenteriae* 1.1869. The reduced amount of SSE1 used in cocktail combinations decreased the rate of biofilm removal, which also indicated that SSE1 was the most effective at killing *S. dysenteriae* 1.1869, further reflecting phage specificity.

The host range of phage SSE1 showed that it could also infect *S. baumannii* 1.10618 [22]. Therefore, the phage cocktails described above were used to remove biofilms (Fig. 2b). The effect of SSE1 alone was consistent with that of the cocktail, with relative removal efficiencies of 33.75%, 32.07%, 33.08%, and 35.21%. This showed that the combination of phages SSE1, SGF2, and SGF3 was synergistic in the removal of *S. baumannii* 1.10618 biofilms, especially when all three were used together. Therefore, a phage cocktail may be considered for clearance of *S. baumannii* 1.10618 biofilm in the case of a single-phage shortage.

Phage SGF2 achieved approximately 26.22% biofilm removal of the host bacterium *S. flexneri* 1.1868 (Fig. 2c). However, two of the three cocktail combinations containing SGF2 had no effect on biofilms and failed to inhibit bacterial biofilm growth, except for SGF2 + SSE1 (8.08% removal). This indicates that only high doses of phage SGF2 have an effect on *S. flexneri* 1.1868.

Phage SGF3 also showed poor biofilm removal (7.02%; Fig. 2d), which was consistent with its ability to inhibit planktonic bacteria. In contrast, phage SSE1, which can infect *S. flexneri* 1.10599, removed 29.62% of the biofilm when used alone, and the effect of several cocktails was greater than that of SGF3 alone. These results indicate that microphage SGF3 may not suitable for sterilisation alone and could be considered in combination with other phages to ensure that the same sterilisation effect was achieved with low amounts of each phage. This will contribute to alleviating the dosage tension of the phage strain.

### Removal effects of lytic proteins on planktonic bacteria

**Fig. 3:**
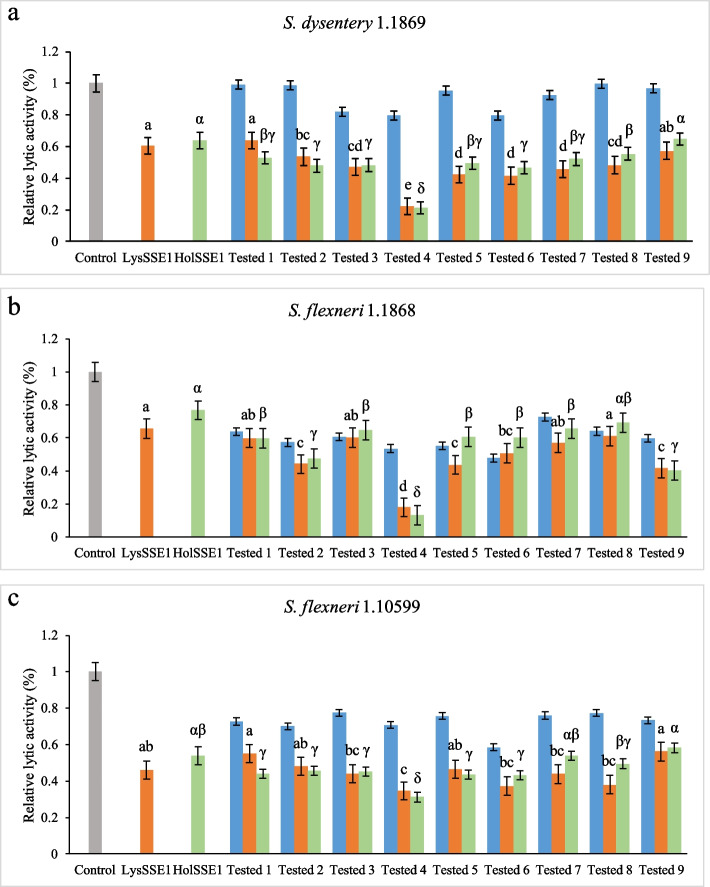
LysSSE1, HolSSE1 and antibiotics were combined to remove planktonic **a** *S. dysentery* 1.1869, **b** *S. flexneri* 1.1868, and** c** *S. flexneri* 1.10599. Gray represents negative control without lytic proteins and antibiotics, orange represents LysSSE1 and antibiotics combination, green represents HolSSE1 and antibiotic combination, and blue represents antibiotic alone. Groups 1, 2, 3, 4, 5, 6, 7, 8, and 9 correspond to erythromycin, gentamicin sulphate, chloramphenicol, cefotaxime, cefoxitin, cephalothin, cardelmycin, tetracycline hydrochloride, and polymyxin b sulphate, respectively. The concentration of the antibiotics used was 1/2 MIC. Error bars represent standard deviation of three biological replicates. The letters a, b, c, d, e, and α, β, γ, δ indicate significant results; the significance level was set at *p* < 0.05

Compared to phages, lytic proteins are not subject to biosafety controversy in sterilisation applications and are safer, in principle. The lysis spectra of the bacteria were expanded for wider applications. As shown in Fig. 3, LysSSE1 and HolSSE1 alone showed different degrees of inhibition of the three *Shigella* strains, greatly increasing the cleavage range compared to the phages. The relative sterilisation efficiencies of LysSSE1 and HolSSE1 alone for *S. dysentery* 1.1869 were 39.6% and 36.2%, respectively, significantly higher than those of each antibiotic alone (Fig. 3a). Both LysSSE1 and HolSSE1 showed synergistic antibacterial effects when combined with antibiotics, significantly enhancing the inhibition of *S. dysentery* 1.1869, with a removal rate almost twice as high as that of antibiotics alone. Cefotaxime showed the strongest synergistic effect with lytic proteins in all experimental groups, reaching removal rates of 77.8% and 78.9% when combined with LysSSE1 and HolSSE1, respectively. This high antibacterial activity is helpful for the prevention and control of *S. dysentery* 1.1869.

Figure 3b shows that the sterilisation rates of LysSSE1 and HolSSE1 alone were not better than those of 1/2 MIC antibiotics for the prevention and control of planktonic *S. flexneri* 1.1868, with removal rates of approximately 34.2% and 23.2%, respectively. The sterilisation rate of cephalothin alone was approximately 52.1%. Considering the cost of sterilisation, it is more economical to use 0.06 µg/mL of cephalothin alone to remove *S. flexneri* 1.1868. However, to achieve a more efficient antibacterial effect, the combination of cefotaxime and lytic proteins would be the best choice, with the strongest synergistic sterilisation efficiency of 86.8%. Based on our results, the combination of HolSSE1 and 0.03 µg/mL cefotaxime was the most efficient method to inhibit *S. flexneri* 1.1868.

As shown in Fig. 3c, the removal of *S. flexneri* 1.10599 with LysSSE1 and HolSSE1 alone was significantly better than that with the individual antibiotics. The antibacterial rates of LysSSE1 and HolSSE1 alone were 53.9% and 46.2%, respectively, and their combination with antibiotics showed synergistic sterilising effects accompanied by a significant increase in the antibacterial rate. The synergistic effects of LysSSE1 and HolSSE1with cefotaxime were the highest (65.4% and 68.9%, respectively). Thus, HolSSE1 and 0.03 µg/mL cefotaxime can be added simultaneously to control *S. flexneri* 1.10599. This was similar to that observed for *S. dysentery* 1.1869 strain. According to the inhibition of these three strains, the antibiotics with the best synergism with lytic proteins all belonged to the β- lactam antibiotics with action sites in the cell wall. This may be related to the sterilisation mechanism of lytic proteins, which have destructive effects on the extracellular membrane [27].

### Removal effects of lytic proteins on single bacterial biofilm

**Fig. 4:**
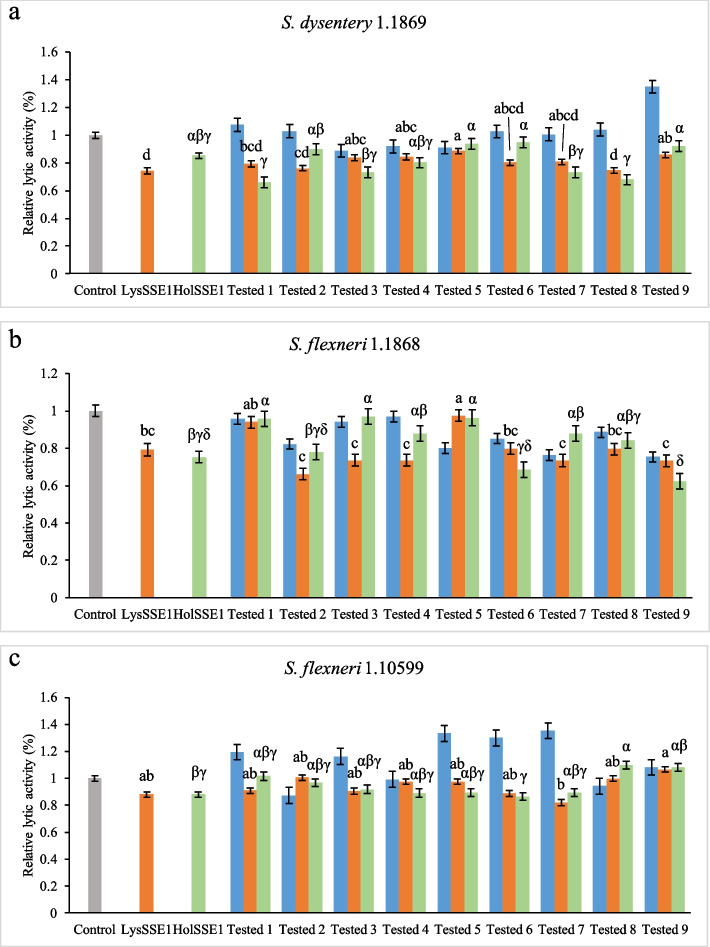
LysSSE1, HolSSE1 and antibiotics were combined to remove the biofilms of **a** *S. dysentery* 1.1869,** b** *S. flexneri* 1.1868, and** c** *S. flexneri* 1.10599. Gray represents negative control without lytic proteins and antibiotics, orange represents LysSSE1 and antibiotics combination, green represents HolSSE1 and antibiotic combination, and blue represents antibiotic alone. Groups 1, 2, 3, 4, 5, 6, 7, 8, and 9 correspond to erythromycin, gentamicin sulphate, chloramphenicol, cefotaxime, cefoxitin, cephalothin, cardelmycin, tetracycline hydrochloride, and polymyxin b sulphate, respectively. The concentration of all the antibiotics used was 1/2 MIC. Error bars represent standard deviation of three biological replicates. The letters a, b, c, d, and α, β, γ, δ indicate significant results; the significance level was set at *p* < 0.05

The effects of the lytic proteins on bacterial biofilms were determined using 96-well polystyrene plates. The experimental subjects included three *Shigella* and two *S. aureus* strains. As shown in Fig. 4, LysSSE1 and HolSSE1 acted individually to remove bacterial biofilms formed on polystyrene surfaces. The combination of lytic proteins and antibiotics to inhibit *Shigella* biofilms showed that the proteins with certain antibiotic would have better synergy and an improved biofilm removal effect. The removal rates of LysSSE1 and HolSSE1 of *S. dysentery* 1.1869 biofilms were 25.78% and 14.84%, respectively (Fig. 4a). None of the 1/2 MIC of erythromycin, gentamicin sulphate, cephalothin, cardelmycin, tetracycline hydrochloride, or polymyxin B sulphate had any effect on biofilms. Among these antibiotics, chloramphenicol showed the best effect, with an efficiency of only 11%. This demonstrated that the stability of the biofilm hindered the removal more than the planktonic bacteria alone. Antibiotics alone must be administered at higher concentrations, which is likely to cause drug contamination and bacterial resistance. This problem can be addressed using lytic proteins and antibiotics to ensure better removal at low drug concentrations. The two lytic proteins had a good effect with erythromycin and tetracycline hydrochloride; particularly, HolSSE1 and erythromycin were used together removed 34% of *S. dysentery* 1.1869 biofilm, and this was the optimal combination for all tested groups. Therefore, the removal of *S. dysentery* 1.1869 biofilms could be improved by combining HolSSE1 and 0.06 µg/mL erythromycin.

For the biofilm of *S. flexneri* 1.1868 (Fig. 4b), LysSSE1 and HolSSE1 removed 20.7% and 24.7%, respectively. However, the effects of antibiotics alone were generally sub-optimal. Similarly to the findings on *S. dysentery* 1.1869 biofilm, removal was enhanced when lytic proteins and antibiotics were combined. LysSSE1 and gentamicin sulphate, LysSSE1 and chloramphenicol, LysSSE1 and cefotaxime, LysSSE1 and cardelmycin, LysSSE1 and polymyxin b sulphate, HolSSE1 and cephalothin, and HolSSE1 and polymyxin b sulphate showed higher efficiency than the lytic protein alone. The removal rates of LysSSE1 and gentamicin sulphate and HolSSE1 and polymyxin b sulphate were 33.7% and 37.8%, respectively, which could be used as an improved method to remove *S. flexneri* 1.1868 biofilms.

As shown in Fig. 4c, the removal rate of *S. flexneri* 1.10599 biofilm by LysSSE1 and HolSSE1 alone was approximately 12.0%, and the effect of the antibiotics was not strong. Except for gentamicin sulphate, the antibiotics had no removal effects. The removal rates in the combinations were similarly weak, at 18% and 13.5% for LysSSE1 and cardelmycin and HolSSE1 and cephalothin, respectively. Therefore, LysSSE1 and cardelmycin were the most efficient combinations, with higher removal efficiencies than those of lytic proteins alone.

**Fig. 5:**
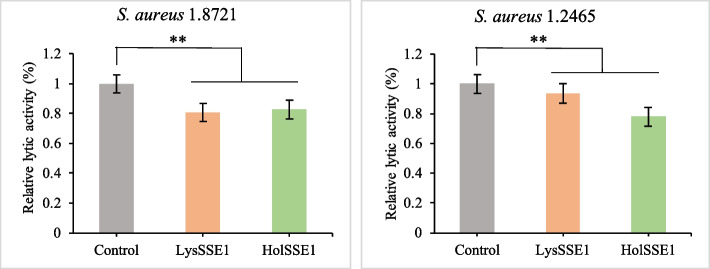
LysSSE1 and HolSSE1 were used to remove the biofilms of *S. aureus*. Gray represented negative controls without lytic proteins, orange for the LysSSE1 added group and green for the HolSSE1 added group. Error bars represented standard deviation of three biological replicates. Statistical analysis was performed using a paired-matched sample t-test, and the double asterisks indicated *p* < 0.01

A previous study found that two lytic proteins could also infect Gram-positive bacteria [25]; therefore, two other *S. aureus* strains (*S. aureus* 1.8721 and *S. aureus* 1.2465) were selected in the lysis spectrum analysis for biofilm culture and removal. As shown in Fig. 5, LysSSE1 removed 19.3% and 6.5% of the two *S. aureus* biofilms, and HolSSE1 removed 17.3% and 22.1%, respectively.

### Removal effects of lytic proteins on multiplex bacterial biofilm

**Fig. 6:**
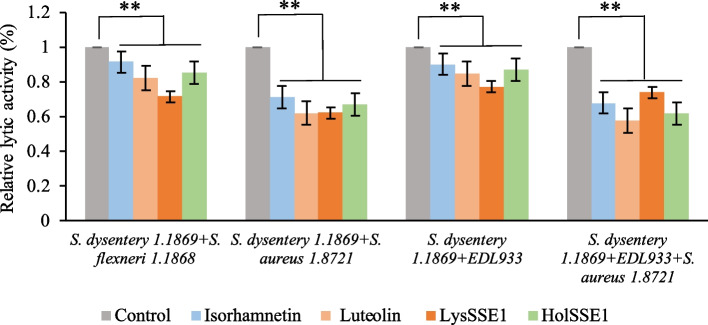
The removal effects of multiple bacterial biofilms. The concentration of isorhamnetin and luteolin was 20 mg/L. Error bars represent standard deviation of three biological replicates. Statistical analysis was performed using a paired sample t-test, and double asterisks indicate *p* < 0.01

Owing to the large variety of bacteria in the environment, more than one bacterial strain is usually attached to an object’s surface. The removal of multiple biofilms is important for environmental safety and human health. LysSSE1 and HolSSE1 can infect many bacteria; therefore, studying the removal of multiple bacterial biofilms would contribute greatly to further applications. Studies have shown that isorhamnetin and luteolin have broad-spectrum antibacterial effects and can disrupt bacterial biofilm [28, 29]. However, the production process is extremely long, and the concentration of the drugs used is difficult to control. As natural substances with a wide lysis spectrum, the lytic proteins isorhamnetin and luteolin were used individually for the removal of multiple bacterial biofilms to compare the application value of lytic proteins. As shown in Fig. 6, four bacterial biofilms of two different types, *Shigella*, *Shigella* and *S. aureus*; *Shigella* and *E. coli*; and *Shigella*, *E. coli* and *S. aureus*, were cultivated separately. The results showed that both LysSSE1 and HolSSE1 exhibited removal effects on multiple bacterial biofilms, and some were better than the effects of isorhamnetin or luteolin. The relative lytic activities of LysSSE1 in the first three biofilms were 28.6%, 38%, and 22.9%, respectively. HolSSE1 removed 38.3% of multiple bacterial biofilms formed by *S. dysentery 1.1869*, *EDL933* and *S. aureus* 1.8721. Therefore, lytic proteins could replace isorhamnetin and luteolin as novel antibiofilm agents.

## Discussion

### Bacterial resistance needs to be urgently addressed

The prevention and control of pathogenic bacteria and bacterial biofilms are hot topics in many fields. Bacterial resistance within biofilms is much higher than that of their planktonic counterparts and is more difficult to control. Furthermore, when multidrug-resistant bacteria form biofilms, the chances of successfully eliminating them are further decreased. High doses of antibiotics may have an effect; however, if improperly applied, these agents can easily cause secondary contamination and contribute to drug-resistant strains that are difficult to kill. Therefore, it is necessary to find alternatives to antimicrobial drugs. Phages and their genetically encoded lytic proteins are promising candidates. Their bactericidal specificity, low susceptibility to resistant bacteria, and environmental friendliness have great potential for the development of novel antibacterial agents.

### Synergistic effect of phages, lytic proteins and antibiotics in removal of planktonic bacteria

The three phages, SSE1, SGF2, and SGF3, were more effective against planktonic bacteria than 1/2 MIC of antibiotics alone. A smart choice for phage cocktails is the use of phages targeting different host structures (phage receptcors or phage targets). The receptors for these hosts are currently unknown, but the phage cocktail results suggest that these phages are good candidates for bacterial eradication, and subsequent studies identifying the receptors are required. Sobhy et al. [30] isolated three novel lytic phages from sewage samples; their cocktail completely inhibited the growth of multidrug-resistant *S. Enteritidis* in vitro. Sun et al. [31] modified phage M13 by inserting a peptide sequence with a high affinity for *Pseudomonas aeruginosa* to enhance biofilm attachment and remove biofilm-containing pathogens more effectively. Phage cocktails have promising applications in combating multi-drug resistant bacteria and biofilms, with a focus on the species diversity of phages and their rational modification.

When the phage was combined with antibiotics, it exhibited a synergistic effect to improve the removal rate of host bacteria. Phage SSE1 had the best effect in combination with cefotaxime. The optimal combination for SGF2 was cefoxitin, with a removal rate of 85.1%. But for microphage SGF3, the sterilizing effect was weak, reflecting the variability between microphages and double-stranded DNA phages in their ability to infect bacteria.

The lysis spectrum of the encoded lytic protein was much wider than that of the phage sterilisation. Their efficacy in killing the three planktonic *Shigella* strains confirms this finding. The removal rates of *S. dysentery* 1.1869 by LysSSE1 and HolSSE1 were significantly higher than those by antibiotics alone. Both lytic proteins exhibited synergistic bactericidal effects when combined with antibiotics, significantly enhancing the removal of *S. dysentery* 1.1869. The strongest synergy was observed with cefotaxime (78.9%), which was higher than that between SSE1 and cefotaxime. Similarly, for *S. flexneri* 1.1868 and *S. flexneri* 1.10599, cefotaxime combined with LysSSE1 or HolSSE1 showed the strongest synergistic sterilisation effect. Thus, the best synergistic antibiotics with phages and lytic proteins all belonged to β- lactam antibiotics, which blocked mucotide productions and hinder cell wall synthesis [32].

### Synergy of lytic proteins with antibiotics and their biofilm removal potential

Some antibiotics at 1/2 MIC had no effect on the bacterial biofilm, demonstrating that biofilms were more difficult to remove than planktonic bacteria. Thus, the advantages of phages and lytic proteins are even more prominent. Studies have confirmed that a phage cocktail can be used to remove *Shigella* biofilms in case of a single-phage shortage. Microphage SGF3 was not ideal for biofilm removal. It is recommended to consider combining SGF3 with other phages to divide the phage dosage and to ensure that the same sterilisation effect is achieved with a low dosage of each phage. Lytic proteins acted better on the biofilm than antibiotics at 1/2 MIC. For *S. dysentery* 1.1869, lytic proteins combined with erythromycin and tetracycline hydrochloride improved the synergistic removal of bacterial biofilms. HolSSE1 and erythromycin were the optimal combinations for all experimental groups. For the biofilm of *S. flexneri* 1.1868, the two combinations of LysSSE1 + gentamicin sulphate and HolSSE1 + polymyxin B sulphate performed the best. LysSSE1 + cardelmycin and HolSSE1 + cephalothin showed better removal of *S. flexneri* 1.10599 biofilm. Lytic proteins cooperated with different classes of antibiotics for the removal of different bacterial biofilms. However, cephalosporins and lytic proteins exhibited synergistic effects when used together. LysSSE1 and HolSSE1 also removed the biofilms of two *S. aureus* strains, CGMCC 1.8721 and CGMCC 1.2465. The potential for lytic proteins to be developed as biological inhibitors is greater than that of phages, which are more widely bactericidal in vitro. In addition, both LysSSE1 and HolSSE1 could remove biofilms generated by multiple bacteria (Gram-positive and Gram-negative bacteria). Some sterilisation combinations performed better than isorhamnetin or luteolin, and could be considered as a replacement.

### The advantage of developing phages, proteins combined with β- lactam antibiotics as novel antibacterial agents

Currently, the use of antibiotics to control bacterial pollution has fallen into a bottleneck, and phages and lytic proteins are undoubtedly a new breakthrough. Although the sterilising effect of SGF3 was relatively weak, it can be used along with other phages. In addition, its short gene length and fewer coding sequences (CDS) provided a great advantage in its design as a probe, and have been more suitable for studies such as detecting pathogenic bacteria in the environment [33]. Compared to SGF3, SSE1 and SGF2 contains complete lytic protein-encoding genes with outstanding sterilisation effects. This indirectly reflects that sequence integrity and the presence of lytic proteins could affect the bactericidal properties of phages. A greater sterilising potential was exploited when phages were combined with antibiotics, partly because of a series of internal differences in their mechanisms of action, which could amplify the antimicrobial effects. Similar synergistic effects were observed for lytic proteins. Overall, β- lactam antibiotics with sites of action located on the bacterial cell wall were the best synergistic type. The similarity in the site of action may have promoted bactericidal effects.

Bacterial drug resistance can be effectively addressed by constantly screening diverse phages based on the bactericidal specificity of the phages and their lytic proteins. This is also due to the endless diversity of phages [34]. In contrast, lytic proteins have a bactericidal advantage over phages because they are not restricted by the host. The bactericidal processes of some phages require the synergistic action of holins and endolysins. Holin disrupts the cell membrane of host bacteria, allowing endolysin to reach the peptidoglycan layer and exhibit its activity [35]. However, this phenomenon is not applicable to all phages, and effective sterilisation was achieved without the participation of holin. Single lytic proteins show a broad bactericidal spectrum in vitro [36]. Effective combinations of phages, proteins, and antibiotics can overcome bactericidal defects and provide new strategies for the prevention and control of pathogens.

## Conclusions

This study demonstrated that phages and their lytic proteins were more effective than antibiotics in killing bacteria and their biofilms. Combined with appropriate antibiotics, they had better sterilisation effects. The combination of β- lactam antibiotics with phage and lytic protein produced a high synergistic effect, which might be related to their mechanism of action. Our results provide a new method for effective bacterial prevention and control. Both the phage cocktail and lytic proteins can be used for biofilm removal. Lytic proteins can inhibit biofilms formed by a variety of individual bacteria and multiple bacterial biofilms and are more widely applied than phages. They can produce a synergistic effect when combined with antibiotics, thereby improving their bactericidal effects. In conclusion, phages and lytic proteins have a high application value in killing *Shigella* and controlling single and multiple biofilms of *Shigella*, *S. aureus*, and *E. coli*. Thus, they have the potential to replace commercial antibiotics in the development of novel antimicrobial agents. A rational combination of phages, proteins, and antibiotics can be used to control specific antibiotic-resistant bacteria. This will play a significant role in reducing antibiotic concentrations and bacterial resistance.

## Data Availability

The datasets used and/or analyzed during the current study are available from the corresponding author on reasonable request.
